# Towards ontology-driven navigation of the lipid *bibliosphere*

**DOI:** 10.1186/1471-2105-9-S1-S5

**Published:** 2008-02-13

**Authors:** Christopher JO Baker, Rajaraman Kanagasabai, Wee Tiong Ang, Anitha Veeramani, Hong-Sang Low, Markus R Wenk

**Affiliations:** 1 Department of Data Mining, Institute for Infocomm Research, 21 Heng Mui Keng Terrace, Singapore 119613; 2 Department of Biochemistry and Department of Biological Sciences, Centre for Life Sciences, National University of Singapore, Singapore 119260

## Abstract

**Background:**

The indexing of scientific literature and content is a relevant and contemporary requirement within life science information systems. Navigating information available in legacy formats continues to be a challenge both in enterprise and academic domains. The emergence of semantic web technologies and their fusion with artificial intelligence techniques has provided a new toolkit with which to address these data integration challenges. In the emerging field of lipidomics such navigation challenges are barriers to the translation of scientific results into actionable knowledge, critical to the treatment of diseases such as Alzheimer's syndrome, Mycobacterium infections and cancer.

**Results:**

We present a literature-driven workflow involving document delivery and natural language processing steps generating tagged sentences containing lipid, protein and disease names, which are instantiated to custom designed lipid ontology. We describe the design challenges in capturing lipid nomenclature, the mandate of the ontology and its role as query model in the navigation of the lipid *bibliosphere*. We illustrate the extent of the description logic-based A-box query capability provided by the instantiated ontology using a graphical query composer to query sentences describing lipid-protein and lipid-disease correlations.

**Conclusion:**

As scientists accept the need to readjust the manner in which we search for information and derive knowledge we illustrate a system that can constrain the literature explosion and knowledge navigation problems. Specifically we have focussed on solving this challenge for lipidomics researchers who have to deal with the lack of standardized vocabulary, differing classification schemes, and a wide array of synonyms before being able to derive scientific insights. The use of the OWL-DL variant of the Web Ontology Language (OWL) and description logic reasoning is pivotal in this regard, providing the lipid scientist with advanced query access to the results of text mining algorithms instantiated into the ontology. The visual query paradigm assists in the adoption of this technology.

## Introduction

Lipids and their metabolites have a very crucial role in the biology and cellular functions of many living organisms. Imbalance or abnormality in lipid metabolism often accompanies diseases such as Alzheimer's syndrome, Mycobacterium infections and cancer. In order to attain a better understanding of the role of lipids in physiological processes, scientists have turned to high throughput technologies and system-level approaches to analyze the lipid composition of living organisms, namely Lipidomics. Lipidomics [1] generates a large amount of heterogeneous chemical and biochemical data that must be integrated and analyzed in a systematic manner. Such efforts are, however, hampered by the lack of consistent classification for lipids.

Lipids, unlike their protein counterparts, do not have a systematic classification and nomenclature that is widely adopted by the biomedical research community. To address this problem the IUPAC-IUBMB [2] developed a standardized systematic nomenclature for lipids. The IUPAC lipid nomenclature suffers, however, from several drawbacks. Firstly, it has not gained widespread adoption since the systematic naming of lipids according to their structures can become long and cumbersome. Furthermore the IUPAC naming scheme was often misunderstood by scientists leading to the generation of many pseudo-IUPAC names that are neither chemically or scientifically sound. Since its emergence in 1976, the IUPAC naming scheme has not evolved to accommodate the large number of novel lipid classes that have been discovered in the last 3 decades. In this context different lipid research groups have developed their own classifications of lipids which are usually very narrow and only applicable for a restricted category of lipid. As a result, the same lipid molecule can be classified in many different ways, and be placed under different types of classification hierarchy and many of these classification systems are not scientifically sound and hence, create a lot of problems for systematic analysis of lipids. Furthermore a single lipid can be associated with a plethora of synonyms.

The LIPIDMAPS consortium [3] attempted to resolve this problem by developing a scientifically sound and comprehensive chemical representation system that incorporates a consistent nomenclature. Their system follows IUPAC nomenclature closely yet is extensible to include new lipids without a systematically defined IUPAC name. This classification scheme organizes lipids into well defined categories that cover the major domains of living creatures, namely, the archaea, eukaryotes and prokaryotes as well as the synthetic domain. This is a significant contribution to lipid research, but adoption of this standard by the scientific community has been gradual and many research groups still use the synonyms or old names. While LIPIDMAPS is scientifically robust it is also a cumbersome naming scheme. The naming of new lipids requires trained experts and subsequent acceptance of new names by members of the lipid community which slows the rate at which novel lipids can be added into the hierarchy. In parallel the arrival of lipidomics has resulted in the discovery of many novel lipids. Given the limitations of the LIPIDMAPS nomenclature, it cannot keep up with the current rate of new lipid discovery. Consequently, many novel lipids such as mycolic acids are left without a LIPIDMAPS systematic name.

In this context the navigation of lipid resources and publications, the *lipid bibliosphere*, remains a challenge for lipidomics practitioners. Legacy literature resources predominantly contain instances of lipid synonyms not yet linked to the LIPIDMAPS systematic name or to a chemically sound classification. A consistent, machine readable and formal knowledge representation of lipid nomenclature is required as a basis for the implementation and deployment of information systems that support the aggregation and interrogation of lipid resources. Ontologies are vehicles of knowledge representation which conceptualise a domain in terms of concepts, relations, instances and constrains on concepts. They provide a common terminology for a domain, a basis for interoperability between information systems, make the content in information sources explicit and provide an index and query model to repositories of information. Such formalisms have increasingly been deployed in the life science information systems which depend on access to expressive knowledge representations. Clinical and Biological ontologies have been extensively reviewed [4,5] however it is relevant to mention here that few ontologies make any reference to lipids.

In recent years a number of approaches have combined text mining with ontologies, such as the *Gene Ontology *(GO), to annotate database entries with segments of biomedical literature [6]. Additionally, ontology based information retrieval systems apply NLP to link documents to existing ontologies [7,8] enabling targeted abstract document delivery. Other systems employ ontologies with text mining and natural language processing (NLP) pipelines. In particular, Witte *et al *2007 [9] outline distinctions between three approaches which combine ontology and NLP, namely; (i) *ontology-based NLP *where the results of NLP are exported to an ontology, using other external resources for the text processing; (ii) *ontology-driven NLP *which actively uses ontological resources for NLP tasks, requiring that ontologies that hold all the information needed by language analysis algorithms; (iii) *ontological NLP *is an integrated approach: using ontologies as a knowledge base for NLP tasks while also exporting the result of NLP analyses into an ontology which can then support subsequent semantic queries to the ontology using description logic reasoners and A-box reasoning over ontology instances.

Description logics (DL) are knowledge representation languages used to represent the terminological knowledge of domain in a structured and formally well-understood way. The OWL-DL and OWL-Lite sub-languages of the W3C-endorsed Web Ontology Language (OWL) are based on a description logic. Reasoning tasks carried out over such ontologies are primarily to ensure the quality of an ontology, to test whether concepts in an ontology are non-contradictory and to derive implied relations. Description logic systems provide their users with various inference capabilities that deduce implicit knowledge from the explicitly represented knowledge [10] namely: (i) the subsumption algorithm determines subconcept-superconcept relationships (ii) the consistency algorithm determines whether a knowledge base consisting of a set of assertions and a set of terminological axioms is non-contradictory (iii) more recently the instance algorithm determines instance relationships (A-box reasoning). The use of description logic reasoning in life science knowledge discovery is an emerging trend and was recently reviewed by Wolstencroft *et al *[11]. The adoption of reasoning by a wider user base has been assisted by the emergence of prototype graphical query composers [12,13].

## Lipid literature navigation infrastructure

Our work is motivated by the need for uninhibited access to un-ordered lipid information in the scientific literature. Here we present a lipid bibliosphere navigation infrastructure consisting of a lipid ontology and a content acquisition and ontology instantiation pipeline (with primary performance results) delivering lipid related sentences derived by text mining. A visual query tool for interrogating the populated lipid ontology is illustrated.

### Lipid ontology

In the absence of a publicly available ontology describing lipid nomenclature and meta-data we sought to formally represent such knowledge in an interoperable and reusable format. To our knowledge it is the first such lipid ontology. The motivation for our lipid ontology can be summarized as follows: (i) to provide, in a standardized OWL-DL format, a formal framework for the organization, processing and description of information in the emerging fields of lipidomics and lipid biology; (ii) to specify a data model to manage information on lipid molecules, define features and declare appropriate relations to other biochemical entities i.e. proteins, diseases; (iii) to enable the connection of the pre-existing or legacy 'lipid synonyms' found in literature or other databases to the LIPIDMAPS classification system; (iv) to serve as an integration and query model for one or more data warehouses of lipid information; and (v) to serve as a flexible and accessible format for building consensus on a current systematic classification of lipids and lipid nomenclature, which is particularly relevant to the discovery of new lipids and lipid classes that have yet to be systematically named. These are established motivations for the use of ontologies. However the lipid ontology we created was designed with additional application requirements in mind and was not designed exclusively as a lipid domain ontology. In particular the literature specification of the ontology was designed to facilitate the population of lipid related sentences and literature metadata into the ontology as instances to support content aggregation and subsequent navigation of the literature space, with A-box reasoning over indexed sentences. The 'sentence' concept and the full literature specification were critical in this regard.

The Lipid ontology has a total of 672 concepts and 75 properties (Table 1). The ontology is the result of integrating schema components from existing biological database schemas, interviews with laboratory scientists, lipid and text mining experts. Some reuse of the existing LIPIDMAPS schema was made. The ontology includes both hierarchical structures supporting full subsumption taxonomies and a broader conceptual frame with novel relationships for specific domain knowledge. The resources for lipid related terminologies and concepts come from the resources listed below [14-17], see also Table 1. Full details of the ontology conceptualization are described in the methods section along with provisions made for database integration, lipid-protein interactions, lipids-disease correlations, the modelling of lipid synonyms and an outline of the literature specification.

**Table 1:** Current number of Concepts in Lipid Ontology.

**Concept name**	**No. of Concepts**
Biological entity	387
Data Source	1
Diseases	28
Experimental Protocol	41
Functional category	75
Isomer	20
Molecular events	2
Pathways	3
Processes	3
Specification	112
Total number of Concepts	672

Number of concepts is divided into 10 groups of sub-concepts that are subsumed by the respective superclasses.

### System architecture

**Figure 1:**
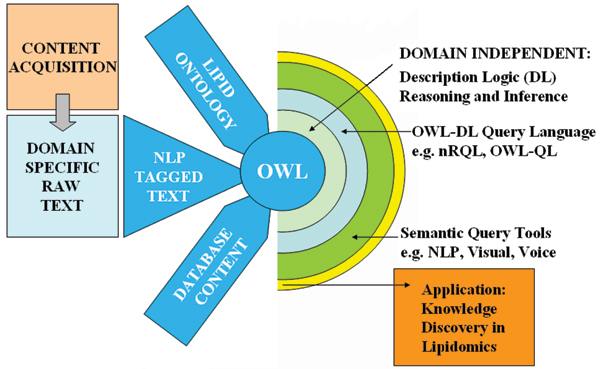
Ontology-centric knowledge-delivery, system architecture.

The system architecture is shown in Figure 1. It consists of a content acquisition engine that drives the delivery of literature. This engine takes user keywords and retrieves full-text research papers from distributed public repositories e.g. USPTO and converts them to a custom format ready for text mining. A workflow of natural-language processing algorithms identifies target concepts or keywords and tags individual sentences according to the terms they contain. Sentences are instantiated (as A-boxes), using a custom designed Java program, to the lipid ontology's literature specification (sentence concept) and relations to instances of each target concept are added into the ontology. The fully instantiated ontology is reasoned over using the reasoning engine RACER [10] and its A-box query language nRQL [11]. A custom built visual query interface, described later, facilitates query navigation over instantiated concept hierarchies, object properties and the visualization of datatype properties in the ontology.

### Literature-driven ontology population

The ontology was populated with sentence instances generated by a content/document acquisition and text mining engine customized to recognize lipid-specific nomenclature and instantiate targeted concepts in the lipid ontology. Sentences tagged by the text mining system are instantiated to the 'sentence' concept of the ontology and relations to instances of the lipids, proteins and disease found in the sentence are created. Instantiated sentences are linked by object properties to instances of the appropriate lipid classes, described in the sentence, in the deep hierarchy of the lipid ontology which comprises of 8 major lipid categories and 352 lipid subclasses.

We provide a preliminary performance analysis of the text processing and ontology population system in assessing the complete lipid-protein interaction mining task. This started with a PubMed literature search for the query "lipid interact* protein" with our content acquisition engine that retrieved 225 search results for the time period September 2006 to April 2007. 110 full-text papers were successfully downloaded. The remaining papers were from journals not subscribed to by our organization, or had no download-able link to the full paper.

**Figure 2:**
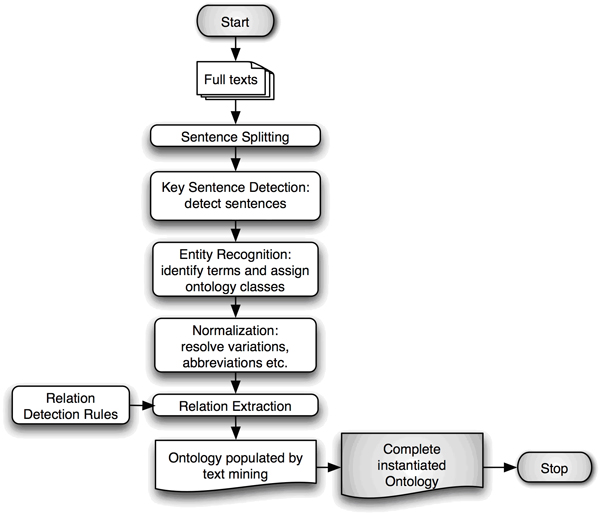
Ontology population workflow.

Entity recognition, normalization and grounding of the full-text documents occurred at a rate of 56 secs/document whereas relation detection 1 second per document.  Ontology instantiation from the entire batch of downloaded papers took 14 seconds without employing rules and 9 seconds with rules using a 3.6 GHz Xeon Linux workstation with 4 processors and 8 GB RAM. A comparison of the ontology population process with and without the relation detection rules (See Figure 2) is described below.

#### 1) Population without relation detection rules

After named entity recognition and relation detection, 23 documents in which no lipid-protein relations were detected were omitted. Ontology instantiation was carried out with the remaining 87 documents. After normalization and grounding, 83 lipid names (68 LipidMAPS systematic names and 15 lipidbank names) and 201 protein names remained. The 68 LipidMAPS names were instantiated into 36 unique classes under the Lipid name hierarchy (at an average of about 2 lipids/class). Based on the hand curated lipid synonym list (See Methods and Named entity recognition) 12 LipidMAPS names were also linked to corresponding KEGG entries. In total 920 sentences describing interactions were detected.

#### 2) Population with relation detection rules

There were 72 documents that had at least one lipid-protein interaction sentence. Normalization and grounding of entities resulted in 68 lipid names (43 LipidMAPS systematic names and 8 lipidbank names) and 176 protein names. The 43 LipidMAPS names were instantiated into 24 unique classes under the Lipid name hierarchy. In total, 465 interaction sentences were detected and manual browsing of the populated ontology indicated that there were fewer false positives than without the use of rules. Currently we are building a corpus to evaluate this more rigorously.

### Navigation of the instantiated lipid ontology

The processing pipeline results in a rich OWL-DL knowledge-base instantiated with text segments. Typically such a knowledgebase is interrogated using highly expressive description logic (DL) query languages that have complex syntactic query language requirements that are not suitable for domain experts [20]. To facilitate the lipidomic practitioner to navigate the relations between lipids, proteins and disease that are identified by text mining we developed an intelligent query interface which reformulates expressive pictorial representations of queries into the corresponding syntax of an A-box query language,  nRQL for issue to the OWL-DL reasoner RACER.

The knowledge navigator query tool (Knowlegtor) receives OWL-DL ontologies as input and passes them to RACER, after which it enters into a dialogue with RACER and issues a series of commands to query elementary features of the ontology for visual representation in the components panel of the tool. The navigator consists of three main panels, a Components panel, the Editor panel and the Output panel Figure 2. The Components panel renders the ontology as a tree structure showing concepts, roles and instances. Each concept is pre-queried to retrieve the number of instances it represents and details of object-properties are determined. Furthermore the Components panel allows drag and drop functionality for query formulation. The Editor Panel is structured as a tabbed pane providing rapid switching between groups of functionalities. The 'Ask a Question' tab contains the query canvas where questions can be formulated by dragging and dropping an element from the tree structure in the Component panel. Each dropped item is associated with an automatically formulated nRQL query. Dragging a single concept invokes the retrieval of all the individuals of a particular concept. Likewise dragging a named relation (object property) queries for the instances specified in the domain and range of the object property. In the query canvas a complex query is built by extending simpler queries through 'right click' enabled instantiated-object property lookup. A separate tab 'Get the Answer' shows a query result in tabular form. In the bottom panel the full text of a sentence is rendered. In addition to facilitating nested role queries (relations) through domain-property-range expansion the knowledge navigator facilitates the identification of the path of (instantiated) relations between any two concepts dragged to the canvas. This provides users with no prior domain knowledge with an additional entry point to building graphical queries which can be subsequently customized. This is achieved using a subroutine which mines the results of iterative nRQL role queries to the ontology until a path(s) is found between the two starting concepts. The knowledge navigator is a generic tool that can support navigation of any OWL-DL instantiated ontology.

## Discussion

The challenge in our lipidomics scenario is the navigation of large volumes of complex biological knowledge typically accessible only in legacy unstructured full-text format. This was achieved through the coordination of distributed literature sources, natural language processing, ontology development, automated ontology instantiation [21], visual query guided reasoning over OWL-DL A-boxes [13]. The major innovations were to: translate the results of natural language processing to instances of a ontology domain model designed by end users; exploit the utility of A-box reasoning to facilitate knowledge discovery through the navigation of instantiated ontologies and thereby enable scientists to identify the importance of newly identified lipids through their known associations and interactions with classes of protein and diseases. An elementary yet crucial component in providing a fully deployable solution to discovery scientists was the provision of sufficient cognitive support for ontology navigation fused with powerful DL-query access allowing the end user to effectively interrogate OWL knowledge resources. Typically end users are well served, by the richly expressive query model provided by the OWL ontology but the A-box query functionality was pivotal to the facilitation of knowledge navigation and this is the major contribution of the knowledge navigation tool, Knowlegator.

Knowlegator was designed to translate user input into query syntax for submission to a reasoner. Specific technical challenges in this regard were: (i) the design of a graph-based query formalism for representing and querying an ontology; (ii) the provision of an adaptive interface providing user interaction with different representations of the ontology which use a consistent query formulation ideology; (iii) provision of a translator to convert a graph drawn on the graph canvas to a well-formed and syntactically expressive DL-query language and in our case, *nRQL*; and (iv) the construction and management of a query syntax formulator that enables users to build queries incrementally, giving instantaneous feedback with every single user action. The syntax formulator facilitates complex queries based on multiple triples found in a graph and their connection is based on whether each domain and range in different predicates have similar properties. The syntax formulator also directs both the translation of graph triples into *nRQL *query atoms triggered by a series of drag 'n' drop and joining actions and the submission of the formulated query to the reasoning engine followed by formatting of the results returned.

**Figure 3:**
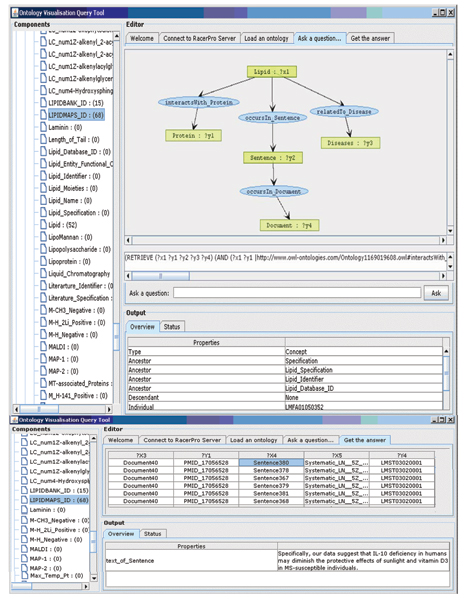
Query Interface of Knowledge Navigator Tool.

**Figure 4:**
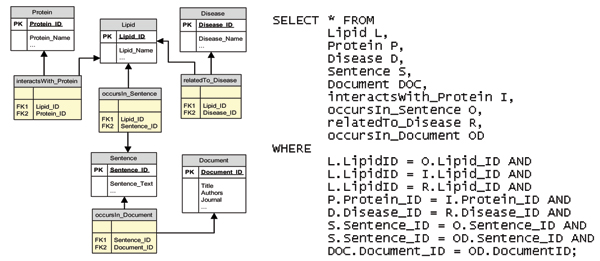
Relational Database Query for Lipid, Protein, Disease Sentences.

We further comment on the enhancement provided by the ontology-centric visual query paradigm provided by Knowlegator by describing an equivalent query to a relational database. For example querying for "lipids that interact with proteins, which occur in a particular sentence of a particular document that are at the same time related to a particular disease" (Figure 3) can be more easily formulated from the ontology by visual query to the ontology than in the relational database scenario. In the database scenario, to process this query each concept should be modelled into a separate table and the relations modelled into additional connection tables to reduce redundancies. Every time there is a new relation, there must be a new relationship table. The SQL query (Figure 4) for the mentioned statement would require 8 table joins and is not particularly intuitive to a user with no prior knowledge of the database. Using the knowledge navigator, the statement can be easily retrieved through a series of right mouse clicks and selecting the required options. This simplification of the 'query issue' action places knowledge discovery back into the hands of domain experts who are sufficiently skilled to appreciate the relevance of the returned results. We hereby remove the need for advanced SQL proficiency by domain experts.

## Conclusion

As the rate of literature accumulation increases we are collectively required to readjust the manner in which we search for information, both with respect to legacy data, heterogeneous formats and on the fly computational results. Often the knowledge 'seeker' has limited knowledge of the content and structure of the resources we search. Here we have illustrated a system that can constrain the literature explosion and knowledge navigation problems. Specifically we have focussed on solving this challenge for lipidomics researchers. In this domain we have to deal with the lack of standardized vocabulary, differing classification schemes, and an array of synonyms which can be linked to the LIPIDMAPS systematic names. In light of these challenges our text mining and visual query tools are able to deliver relevant content and precise access to content. Our ongoing work investigates the inclusion of the chemical structure descriptor InChI as the unit of lipid instances as well as the scale up of our platform for the analysis of larger volumes of literature with greater recognition of legacy lipid terms.

## Methods

### Ontology development

Our goal was to take advantage of the combination of the OWL [15] framework with expressive Description Logics (DL) without losing computational completeness and decidability of reasoning systems. We used Protégé 2000 [23] as a knowledge representation editor. The Ontology was designed with a high level of granularity and implemented in the OWL-DL language. During the knowledge acquisition and data integration phase of ontology development, we consulted lipid content in the form of database annotations, texts from the scientific literature, and entries within distributed biological databases.

Information about individual lipid molecules was modelled in the Lipid and Lipid Specification concepts. The Lipid concept is a sub-concept of Small_Molecules subsumed by the super-concept of Biomolecules. Under the Lipid concept we include the LIPIDMAPS systematic classification hierarchy. The hierarchy currently consists of 8 major lipid categories and in total has about 352 lipid subclasses. The LIPIDMAPS systematic name is modelled as an instance of a lipid. The use of the LIPIDMAPS systematic name enables the linkage of the LIPIDMAPS classification system to other lipid associated information found under Lipid_Specification concept and the rest of the ontology. The Lipid_Specification is a super-concept representing information about individual lipids (Table 2). The Lipid_Specification concept entails the following sub-concepts; Biological_Origin, Data_Specification (with a focus on high throughput data from Lipidomics), Experimental_Data (mainly mass spectrometry data values of lipids), Properties, Structural_Specification and Lipid_Identifier (that carries within it 2 other sub-concepts; Lipid_Database_ID and Lipid_Name).

**Table 2:** Relationship between Lipid sub-concept and other sub-concepts under Lipid_Specification.

**Domain**	**Property**	**Range**
Lipid	hasBiological_Origin	Biological_Origin
Lipid	hasData_Specification	Data_Specification
Lipid	hasExperimental_Data	Experimental_Data
Lipid	hasLipid_Identifier	Lipid_Identifier
Lipid	hasProperties	Properties
Lipid	hasStructural_Specification	Structural_Specification

### Provision for database integration

To facilitate data integration each Lipid instance is related to other databases with the has_DatabaseIdentifier property (Table 3). The object property has_DatabaseIdentifier links a lipid individual to a database identifier. Specifically, our lipid ontology is designed to capture database information from the following databases: Swiss-prot, NCBI OMIM and PubMed, BRENDA and KEGG. Moreover, we have also made provisions in the ontology for it to store information from NCBI taxonomy database. The database record identifiers from each database are considered as instances of the respective database record. Identifier concepts are subsumed by a database specific superclass. For example, the Swiss-Prot_ID concept is subsumed by the Protein_Identifier super-concept which is in turn subsumed by the Protein_Specification super-concept. The presence of a Protein_Specification super-concept is provisional, should we decide to enrich the ontology with protein related information.

**Table 3:** Relationship between Lipid sub-concept and other sub-concepts that relates to external databases.

**Domain**	**Property**	**Range**	**Database source**
Lipid	hasSwiss-Prot_ID	Swiss-Prot_ID	Swiss-Prot
Lipid	hasOMIM_ID	OMIM_ID	OMIM
Lipid	hasEC_num	EC_num	BRENDA
Lipid	hasKEGG_ID	KEGG_ID	KEGG
Lipid	hasPMID	PMID	PUBMED

### Lipid-protein interactions

The inclusion of Lipid protein interactions in the ontology necessitates the existence of the concept Protein which is subsumed by Macromolecule and Biomolecule concepts. The systematic name of a protein in the Swiss-Prot database serves as an instance of the Protein concept. Lipid instance is related to a protein instance by the object property InteractsWith_Protein.

### Lipids and disease

Information about lipids implicated in disease can also be modeled. We added a primitive concept of Disease in the ontology. A disease name is considered as a disease instance which is related to a lipid instance by the object property hasRole_in_Disease property.

### Modelling lipid synonyms

Due to the inattentive use of systematic lipid classifications, a lipid molecule can have many synonyms which need to be modelled into the ontology. In our Lipid Ontology, a lipid instance is a LIPIDMAPS systematic name and synonyms include the IUPAC names, lipid symbols and other commonly used lipid names (both scientific and un-scientific ones). We address the multiple name issue by introducing two sub-concepts, IUPAC and Common. These two concepts are sub-concepts of Lipid_Identifier, which is subsumed by the super-concept Lipid_Specification. For every LIPIDMAPS systematic name, there is typically one IUPAC name and one or more common names. Every LIPIDMAPS systematic name can be related to an IUPAC name via hasIUPAC property and to common names via hasCommon_Name property. A common name is related to an IUPAC name via a hasIUPAC_synonym property. In the same way, the IUPAC name is related to the common name via a hasCommon_synonym property. Lastly, the common name and IUPAC name are related to the systematic name via a hasLIPIDMAPS_synonym property. The current ontology modelling does not account for a common name that has other common names as its synonyms, i.e a direct synonym relationship between 2 common names. In order to identify this type of relation we have to deduce such relationship in an indirect manner. Where a common name is related to a systematic name, the systematic name can be examined for common names. As long as there is more than one common name found linked to the systematic name, we can be certain that these common names are synonyms of one another.

The current ontology also does not yet address the presence of broad synonyms, where a broad synonym is a name that is general enough to describe several lipid individuals. By example, when several systematic names or IUPAC names are related to a common name, that common name is in fact a broad synonym.

### Literature specification

One of the main applications of Lipid Ontology is to provide a knowledge framework where effective text-mining of lipid related information can be carried out. To achieve this, we introduce a top level Literature_Specification super concept into the ontology so that non-biological units of information can be instantiated. The Literature_Specification comprises of 10 sub-concepts, namely Author, Document, Issue, Journal, Literature_Identifier (with a sub-concept PMID, the PubMedIdentifier), Sentence, Title, Volume, Year. The Document concept captures details of documents selected by the end user for subsequent text mining. It is related to multiple concepts within the Literature_Specification hierarchy via several object properties. The Document concept also has 3 datatype properties; author_of_Document, journal_of_Document, title_of_Document that become instantiated with the author name, journal name and title of the article in the form of text strings. In future versions we intend to adopt full Dublin Core units of document metadata by importing the OWL-DL version of this ontology and extend it to include our Sentence concept which is related to the concept Document via the occursIn_Document property. Sentence also has a datatype property, 'text_of_Sentence' that is instantiated by a text string from the documents that were found to have a lipid name and a protein name occurring in the same sentence. Sentence is related to Lipid and Protein concepts via the hasLipid and hasProtein object properties.

### Ontology population workflow

In this section we describe the content acquisition, natural language processing and ontology instantiation strategies. Primarily ontology instances are generated from full texts provided by the document delivery system using text mining toolkit called BioText Suite, which performs text processing tasks such as tokenization, part-of-speech tagging, named entity recognition, grounding, and relation mining .

#### Content acquisition

We employ a content acquisition engine that takes user keywords and retrieves full-text research papers. Collections of research papers are converted form their original formats, e.g. html, pdf etc. to ascii text and made ready for text mining by a customized format converter.

#### Named entity recognition

We employ a gazetteer-based approach for entity recognition. We earlier considered well-known NER systems (e.g. [24]) but had to abandon them due to the large number of false positives extracted on our documents. This probably indicates that retraining is required but, in the absence of a corpus for the domain of Lipidomics, we decided to opt for the gazatteer lookup that yields high precision though at a reduced recall.

The gazetteer processes retrieved full-text documents and recognizes entities by matching term lists against the token of a processed text, and tags the terms found. During tagging, the ontology class of the term is added as attribute, and this attribute is used later during the instantiation process to identify the correct ontology class for population. Separate term lists were employed for detecting lipids, proteins and diseases. The lipid name list was generated from Lipid DataWarehouse (Koh and Wenk, unpublished results) containing lipid names from LIPIDMAPS, LipidBank and KEGG. Each lipid name is identified by a systematic name LIPIDMAPS [3], IUPAC name, Common name and optionally other synonyms, along with a database identifier. As of April 2007, LIPIDMAPS contained 10103 entries. There were 2897 LipidBank entries and 749 KEGG entries linked to the corresponding entries in LIPIDMAPS via the database ID. All these linked entries were collapsed and grounded to their respective systematic name. Term lists were created for each category of names: Systematic, IUPAC, Common and Other synonyms. The manually curated Protein name list from Swiss-Prot  was used for grounding of proteins found in literature and further consolidated by combining all canonical names and synonyms. Grounding used the Swiss-Prot ID. A disease term list was created from the Disease Ontology of Centre for Genetic Medicine  and used for grounding disease names.

#### Normalization and grounding

Entities recognized in the previous step need to be normalized and grounded to the canonical names, before instantiation. The protein canonical names are straight forwardly defined as those found in Swiss-Prot. The grounding is done via the Swiss-Prot ID. For lipid names, we define canonical names depending on the originating database: the systematic name is taken as the canonical name for LIPIDMAPS, the LipidBank name for LipidBank and the KEGG name for KEGG. The latter two are defined only if there is no link to the corresponding related entry in LIPIDMAPS. The respective database ID's are used during the grounding step. In cases of names having two or more ID's, the grounding process assigns precedence first to LIPIDMAPS, then LipidBank and finally KEGG. Disease names are grounded via the ULMS (Unified Medical Language System) identifier, UMLS_ID.

#### Relation detection

In this step we identify the Lipid-Protein and Lipid-Disease relations, using the grounded entities. We adopt an association mining approach whereby two entities are said to be related if they co-occur in a sentence. Thus, every document is parsed to extract sentences and then co-occurrence detection is invoked. To reduce false positives, we built a ruleset (42 rules) in consultation with the Lipidomics domain experts and considered only those sentences that matched one of the rules. Primarily the rules looked for the presence of a relation keyword (e.g. 'bind','express', etc.) or its inflected form. The co-occuring Lipid-Protein or Lipid-Disease pairs from the resulting sentences are returned along with the interaction sentences.

#### Ontology population

In this step we collect all the mined knowledge from the previous steps to instantiate the ontology. The grounded entities are instantiated as class instances into the respective ontology classes (as tagged by the gazetteer), and the relations detected are instantiated as Object Property instances. We wrote a custom script using the most-widely used OWL programming framework, JENA API  for this purpose.

## Competing interests

The authors declare that they have no competing interests.

## Authors' contributions

Christopher Baker was Principal Investigator of this initiative. He conceptualized the system architecture and wrote the manuscript. Rajaraman Kanagasabai designed and built the text mining and ontology instantiation pipeline. Wee Tiong Ang built the knowledge navigation tool (Knowlegator), Anitha Veeramani designed and implemented the content acquisition engine. Hong-Sang Low built the lipid ontology. Markus R. Wenk is a lipid domain expert and provided the vision for knowledge discovery in lipidomics
